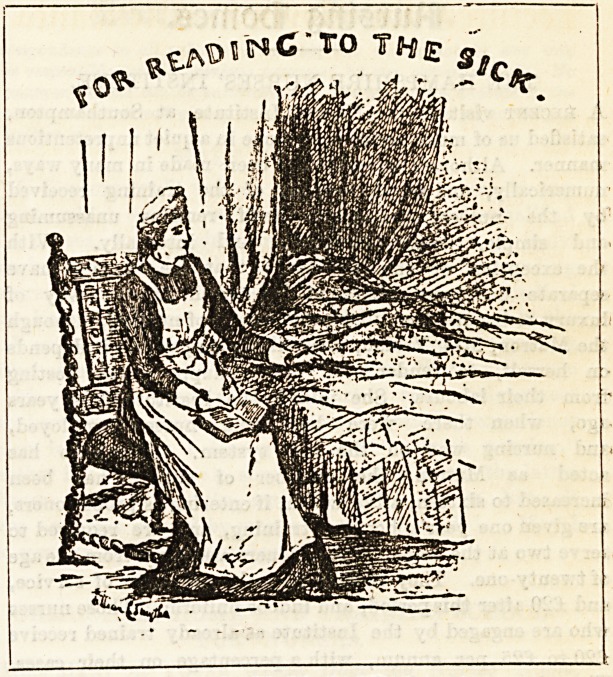# The Hospital Nursing Supplement

**Published:** 1892-09-03

**Authors:** 


### "The Hospital" Nursing Mirror.

Being the Extra Nursing Supplement of "The Hospital" Newspaper.

Contributions for this Supplement should be addressed to the Editor, The Hospital, 140, Strand, London, W.C., and should have the word "Nursing" plainly written in left-hand top corner of the envelope.

### En Passant.

PRACTICAL POINTS.—We have been asked by several correspondents during the last few weeks if we could have a column in which questions could be asked on practical points of management, supply, and so on, such as the necessary supply of linen to a given number of beds, or ideas on food and its quantity, and many other subjects. In answer to our readers, if they will look in the number of this journal for May 14th, p. 112, they will see that a practical point column was started in the "Workshop," which will deal with these points; we shall be very glad to admit any questions of difficulty, and we will ask our readers to answer them from their own experience, so that if a question appeared one week, the answer to it could appear in the following one. Purely nursing questions are answered in "Notes and Queries."

THE FEVER HOSPITALS.—It has been asserted lately that, unless specially asked for, no tidings of patients in the metropolitan hospitals for infectious diseases can be had by the friends and relatives until the patients become convalescent, or decidedly worse. Visitors are not *encouraged* at fever hospitals, and the reason of this is easy to appreciate, but our experience is that an enquiry made at the entrance at any time will elicit reliable and prompt information as to a patient's case. The idea of a place displaying a list and bulletin of each case, hung in a place of easy access, which has been suggested, does not commend itself. How many people would, we wonder, approve of a public notification that one of their household had fever of any sort. We have hitherto found that personal enquiry at the entrance is all that is needed. If this is not so, and some have been specially lucky, then it is a disgrace which needs remedy; but it is a pity to make general statements, as they reflect a disgrace on an entire system when the fault may lie in one hospital only.

FEVER NURSES.—In the course of his address at Nottingham, before the British Medical Association, Dr. H. T. Bulstrode mentioned a fact of considerable interest to nurses. He said that "it was to be feared that in a large number of fever hospitals the nursing was not first-class, and the patients were often entrusted to people who had had little or no special training." This is a point on which we have often discoursed, and it is one which demands very special attention. Instead of shrinking back from the very name of such an institution, nurses would do well to consider themselves incompletely trained until they have gone through a term of work in some place of the kind. Many women, not young girls, but mature and experienced women, could reconcile themselves to what Dr. Bulstrode calls "the social ostracism" for a reasonable period if they once understood that personal risk is reduced to a minimum in hospitals devoted to the treatment of infectious diseases, and the personal value of any nurse is considerably enhanced by practical experience of fever work. The second point of special interest in the addresses on the subject of fevers was the transmission of the patients, and it was specially recommended that intelligent women should be trained and employed as ambulance nurses, in which capacity their tact, as well as their knowledge, might aid in the discovery of other infections in houses, and they could thus assist in the protection of the public to an almost unlimited extent.

A NEW HOME FOR INVALIDS AT CANNES.—Mrs. Dan Norris, R.R.C., well known to many nurses as Miss Rachel Williams, and formerly Lady Superintendent at St. Mary's, Paddington, is going out to Cannes in October to open a home of which many invalids and others will be glad to know. Mrs. Norris will be able to receive invalids who want to winter in the South, and who cannot bear the noise and bustle of hotels; and she has also made arrangements to receive young people of either sex who require medical care and supervision. Her home will also be available for those who are taken ill while travelling, as Mrs. Norris will have the assistance of thoroughly-trained nurses. The charges will include everything excepting medical attendance, night nursing, wine, fire in bed-rooms and lights, and will commence at three guineas a week. In certain cases arrangements will be made to receive a friend or relative as well as the patient. Mrs. Norris, who is at present in London, will be glad to give any further information. Her present address is 2, Dennington Park Mansions, West Hampstead. The name of the new home is Les Agaves, Cannes.

THE PROSPECTS OF SOUTHAMPTON NURSES.—We are very glad to hear that "Queen's Nurses" begin work in this town on October 1st, and that their inauguration promises a new era for nursing of all sorts in Southampton. Public feeling is being brought to bear on the Guardians to provide trained nursing for those receiving both in and out door relief, and we can only hope that Dr. Maclean's proposal will before long come before the Board again, and receive a very different answer to the last, and that the Southampton Guardians' donation will figure among the first list of subscribers to the Q.V.J.T.N. The economy of employing efficient workers needs no comment from us, and we should have thought that the cruelty of giving untrained nurses to the sick had by this time been brought home to everybody, including poor law guardians.

THE KITCHEN AT PLAISTOW, E.—A good many of our readers barely know where Plaistow is; suffice it to say that it is in Essex, and a very much longer way east than many people ever go, and suffering and illness are plentiful there, in the land where the marshes formerly had it all their own way. The man in regular work, whatsoever, is a rich man in Plaistow; the poor man and his family may, perhaps, be left then to imagination. We want to plead for the kitchen at St. Mary's Nurses' Home. Sometimes when the nurses are on their rounds they find they can do no more good for lack of a little simple nourishing food. A kind friend has provided a stove and the necessary utensils, and now we want our readers to see what they can do to provide the materials to cook. So many of our nurse readers have rich friends, and many of our non-professional readers would help, if they knew how to start. Small presents of grocery, or fresh vegetables and fruit, or postal orders for gravy beef, or a couple of fowls out of somebody's poultry yard, are examples of what could be done to start this kitchen supply. The food will be only for the sick folk, and none of it will be wasted; and we do earnestly commend this appeal to our readers, as we know what the nurses' work is doing in Plaistow. A parcel, carriage paid, sent to St. Mary's Nurses' Home, Plaistow, E., will be gratefully acknowledged, and may we remind our readers that "he gives twice who gives quickly"

### Lectures for Asylum Attendants.

By William Harding, M.B.

### I.—INTRODUCTORY (*continued*).

The brain is the organ of the mind. The function of some of its nerve cells is to produce thought, will, consciousness, memory, and this is done by means of the communicating fibres through which messages pass to and fro between the cells in different parts of its surface. Disease or destruction of these nerve cells or of the communicating fibres may reduce the eloquence of the most sagacious statesman to the incoherent chatter of the drivelling lunatic. The healthy brain, by its will power, sets in action and directs the muscles of the body. The surface of the brain is marked out into areas which have special duties assigned to them. One area presides over the movements of the upper limb, another over those of the lower limb. Thus when, by the action of certain cells in another part of the brain, the thought and the will to do a certain action is elaborated, a message, by means of the communicating fibres, is sent to the cells which preside over the muscles necessary to perform the action willed. These cells, by means of the efferent motor fibres, send the order out and the muscles contract.

Certain areas receive and interpret messages from the organs of special sense; if the cells be diseased the message may be misinterpreted, and we may have a perverted sensation; if the cells be destroyed the special sense is lost altogether.

It is a curious fact that the one side of the brain presides over the other side of the body. The fibres that pass between the brain and the two sides of the body cross in the spinal cord, the fibres from the right side of the body going to the left side of the brain and *vice versa.* Thus a hæmorrhage into the right side of the brain may cause paralysis on the left side of the body.

We can now understand what is meant by an idiot, as distinguished from a lunatic. In an idiot there has been an imperfect development of the nerve centres. He can no more be expected to have a sound mind than a man born with stumps for his legs could be expected to play football. His nerve cells are defective, and though by education the greatest possible use may be made of them, yet he can no more reach the average standard of intelligence than a weakling could by training ever hope to rival a Hercules. In both cases the most that the machine is capable of may be attained, but no more.

A lunatic, however, has had an average development to begin with, but his mental powers have become impaired owing to disease or injury.

According to the function of the part of the brain attacked the symptoms of the affection vary. We have seen patients helplessly paralysed but able to converse sensibly. We know cases whose physical powers are as good as our own, but whose minds are an utter blank.

The brain is a delicate structure; slight injuries or structural changes play great havoc with its functions. It is easily affected by the condition of the blood, as is seen in the delirium and convulsions in various diseases.

Our asylums are in a great measure huge lumber rooms for human wreckage, and seem standing reproaches for the inefficacy of our efforts to treat mental disorders. The explanation lies in the delicacy of the nervous tissues. In most diseases some slight permanent change is left in the structure of the organ or tissue affected without materially impairing its functions. In the brain, however, slight changes mean the mental life or death of the patient. Hundreds of cases exist in our large asylums as hopeless lunatics who are as surely cured of the acute affection from which they suffered as are those people who have had pleurisy; are said to have recovered, and are now doing their duty in the world. The difference lies in the importance of the organ affected. In the one case, even a considerable amount of structural change may be a matter of comparative unimportance; in the other, a slight change means all the difference between a useful life outside and a living death within the walls of an asylum.

Any portion of the nervous system may be diseased or injured, and the symptoms will vary in character and in gravity, according to the nature and importance of the function of the part affected. If a sensory nerve be divided, then sensibility is lost in the area supplied by that nerve; if a motor nerve be divided, the muscles' supplied by that nerve are paralysed. In the one case messages from the ends of the nerve affected cannot pass to the brain, and in the other orders from the brain to the muscles are cut off. Thus the muscles may be healthy, the brain cells working normally, and the track down the spinal cord clear, but there is a block in the nerve which conveys messages to the muscle or from the sensory area. Disease or injury of one of the nerves of the special senses is followed by impairment or loss of the function of the organ or the part of it. Thus affections of the nerves of the eye may be followed by blindness, &c.

If the spinal cord be diseased or injured, some or all of the messages ascending to or descending from the brain will be cut off, and then the brain is isolated from that part of the body below the level of the affection in the spinal cord. No communications can pass upwards if the whole thickness of the cord be implicated, nor can any orders be transmitted downwards. The brain, nerves, and muscles are in good working order, but the stoppage of the messages in the spinal cord causes loss of both sensibility and motion below the injury. If any part of the thickness of the cord be left unaffected, then the messages which pass by that portion of it will still be transmitted.

Disease may affect any part of the brain, and the result will depend upon the function of the part affected. We said that the fibres entering the brain from the spinal cord spread out to reach the various parts of the brain to which they were distributed. These fibres may be injured or destroyed by hæmorrhage, and according to the function of the strands of fibres affected we have paralysis of motion or sensibility over the part of the body with which they are in communication.

We must distinguish between the effects of irritation or undue action of the nerve cells and those which follow upon their destruction. In the former case we have an alteration or exaggeration of the usual work of the cells and in the latter the function over which they presided is utterly lost. Any area of the surface of the brain may be affected. If it be that portion which presides over the muscles of the arm, and the disease has not advanced beyond the stage of irritation we may have spasmodic contractions of the muscles arising from the working of the irritated nerve cells which have got beyond the control of the will. If the cells be completely destroyed then the muscles under their orders are paralysed. Here the whole line is free for efferent messages to be sent out, but owing to the destruction of the nerve cells there are no orders to send. The machine is perfect, but there are no means of putting it into action.

Should a portion of the brain engaged in the interpretation of messages from an organ of special sense be affected, we may have symptoms due either to the irritation or to the destruction of cells in that centre. If we suppose the area for sight to be affected then from irritation and perverted action of the cells the individual may see flashes of light, or may even fancy that objects of a definite shape are before him. Of course, if the cells be destroyed entirely then blindness results. Similarly we have symptoms arising from the affection of areas, concerned with smell, taste, hearing, &c.

(*To be continued.*)

### How to Procure Nurses for India Locally.

SIR,—In Mrs. Robertson's letter, which appeared in the HOSPITAL "Nursing Supplement" of July 9th, she states that nurses from England could utilise their skill for the instruction of local talent. Being an Anglo-Indian myself, and having from time to time come in contact with all grades of that community, I should like to emphasise the statement that India is able to provide good nurses for her-self if means were adopted for training them; if, too, the status of a nurse in India were the same as it is in England, in a few years India would be as proud of her nursing staff as England justly is now. We must look to Anglo-Indians, however, for our nurses, for though the numbers of native women training as midwives is daily increasing, it is very rare to see them training as sick nurses, and, indeed, caste prejudice would prevent them in many ways from being useful nurses, especially in English homes. I have spoken of Anglo-Indians, let it be understood that I include in that term those of purely European parentage, many of whom are well educated girls and women, who would gladly devote their lives to the noble work of nursing if, as it has been suggested, a scheme were started for training nurses for India on a larger scale than has yet been attempted. I shall now mention a few of the sources from which nurses could be obtained. There are many girls educated in England who go out to India to live with friends or relations in out of the way places, such as tea or indigo factories, where for weeks together they do not see a single European, out of the home circle. For a short time, however, the life seems full of interest, especially in the cold weather, when an out-of-door life can be led; then the morning rides and the afternoon tennis or boating or country walks supply all that can be desired in the way of enjoyment and interest, but after a time even this gets monotonous without "something to do." From the numerous schools in all parts of India hundreds of girls who enjoy the advantages of a good education pass out every year. Many of these would swell the numbers of those training at a nursing institute. There are those, too, who have not had the opportunities in the way of education that their sisters enjoy in the present day, but who have a knowledge of home life, and more or less experience in nursing, for there are few homes in India that have not been visited by sickness, involving long and tedious nursing by wife, mother, aunt, or sister. For the nurses already working in some of the large hospitals in Calcutta and other parts of India I have the highest regard. Many of them have been working for over twenty years at their posts, and though they are for the most part "Eurasians and soldiers' wives," their devoted care of their patients, and their quiet, respectful manner, gain for them the admiration of all with whom they come in contact. ADA NIEBEL,

Lady Doctor, Bhopal, India.

London, August 23rd, 1892.

### Appointments.

[It is requested that successful candidates will send a copy of their applications and testimonials, with date of election, to THE EDITOR, The Lodge, Porchester Square, W.]

DUNDEE CONVALESCENT HOME.—The election for the post of Matron at this Home, which took place on August 25th, has resulted in the appointment of Miss Jeanie Bowman.

PADDINGTON INFIRMARY.—Miss Elizabeth Thompson, who was trained at the Preston Infirmary, and has held the post of Charge Nurse for the last six years at the Bristol Royal Infirmary, has been elected Night Sister of the Paddington Infirmary.

ROYAL INFIRMARY, BRISTOL.—Miss Florence Lloyd, who was trained at the General Hospital, Birmingham, and for the last sixteen months has worked in the Diphtheria Ward at St. Bartholomew's Hospital, has been elected Night Sister at this infirmary.

### "LOSS OR GAIN?"

WHETHER a life of suffering is loss or gain to human beings is not to be decided without thought. At first sight it would seem to be miserable indeed to live shut off from all pleasure. "Fixed in a rooted vegetable life," the present full of pain, the happy memories of the past faded or blotted out by agony, and the future a blank or worse. There is no doubt in any mind that health is one of the greatest blessings we can have, it sweetens toil, makes our joys fuller, and our spirits bright, while sickness weakens the body and takes away the edge from all enjoyment. Yet health does not necessarily bring us nearer to God, for how few of us recognise His hand in the numberless mercies we receive, or feel that He directs the soft wind which fans our brow, and that in our social intercourse, when hand joins hand, and heart goes out to heart, that God is in the midst of us. Does the sweet song of the birds, or the lovely landscape give us pleasure because the voice of God's boundless love speaks to us throughout the universe?

Faintly, if ever, do we realise it, but when the sharp strokes of pain run through our flesh we know that God is indeed speaking to us, holding us close as a mother does her babe with constraining, loving touch. It is His finger tips which press upon our throbbing foreheads, they may seem heavy, but the touch is light and careful, and measured to our strength. He stands by us always with unfaltering patience, till at length we grow patient too, and would not miss the anguish which comforts with the assurance that He is ever at our side, by night and day alike we are His care.

"He gives His angels charge of those who sleep; but He Himself watches with those who wake." If suffering is the law of life, let us seek the strength by which to live and suffer.

The world is a fitting school for patience, and while we remain in it we will learn of Christ to bear our Cross manfully, whether it be secret in our own hearts or open to all beholders. Faith and hope in Him will lead us step by step towards the rest from pain and labour in this Paradise of God. Our poor fragile bodies will there be strong and glorious, beautiful in health and vigour that shall last throughout eternity. As we bear with gladness and courage the burdens laid on us here, so will our reward be paid hereafter in endless gifts, and in that blessed place where we shall see God face to face, and know Him as He knows us, in all the brightness of His glory.

### Nursing Homes.

### THE HAMPSHIRE NURSES' INSTITUTE.

A recent visit to the Nurses' Institute, at Southampton, satisfied us of much useful work done in a quiet unpretentious manner. Although advance has been made in many ways, numerically, and in the quality of the training received by the nurses, the home itself remains unassuming and simple, both externally and internally. With the exception of the advantage that the nurses have separate bedrooms, there is not much in the way of luxury to beguile the dillettante votary of nursing, although the Matron, Mrs. Varian, does all she can, where it depends on herself, to render the nurses happy when resting from their labours. She joined the Institute five years ago, when there were but nine nurses employed, and nursing was on the old system. Since she has acted as Matron, the number of nurses has been increased to sixteen, all of whom, if entering as probationers, are given one year's hospital training, and are required to serve two at the home. Probationers are taken from the age of twenty-one. They receive £18 the first year of service, and £20 after this period, and indoor uniform. Those nurses who are engaged by the Institute as already trained receive £20 to £25 per annum, with a percentage on their cases. There is space in the home for twenty nurses, but really competent nurses for nursing homes are not easy to secure, and were more especially difficult to find during the past year. The institution can naturally only afford to train a limited number of probationers, and therefore the desired addition to the staff is at present in abeyance. During the year 110 families were nursed, and over 70 cases refused, a fact that testifies to the popularity of the institution. Besides the private nurses, five of whom are monthly, the institution employs three district nurses. These reside in their own homes, and are of the old-fashioned order of nurse, one having been working thus for twenty years. Doubtless when funds are available, and it is necessary to replace the present employés, nurses with full institution training only will be engaged. It seems to us that in district nursing, above all others, thorough training and experience should be ensured. We would in no wise advocate the usurpation of the doctor's authority by the nurse, but those who visit amongst the poor know full well that there are occasions when the busy parish doctor being unavailable, cases of emergency arise when all the experience and knowledge of a nurse are taxed to the utmost. In cases of doubt as to treatment, the poor cannot and do not think of resorting to the medical man for further instructions, and in these cases the nurse is often able to avert the direful consequences of injudicious actions. In making these remarks we are not in any way reflecting on the efficiency of the district nurses now employed, only to the system, as a principle for future action. The present staff have proved of inestimable benefit to the sick poor, by whom their services are much appreciated. Two hundred and sixty-five cases were attended during the past year, and 6,640 visits were made by the day nurses, whilst the night nurses spent 263 nights at the bedside of the sick. Such a record of work is worthy the highest praise. The Matron stimulates her workers by her example, being a constant visitor in the various parishes, in spite of her responsible duties elsewhere. She has drawn up an excellent code of rules for the guidance of her nurses amongst the poor. These we subjoin, as they may prove of interest to those engaged in the management of like institutions. Three district nurses are none too many when employed over so large an area as Southampton, and leave no margin for work in outlying districts, even when their work is supplemented, as it is, by the private nurses when unemployed. To extend such useful work means increased funds, and as the fees received for private nursing are strictly moderate, the institution is largely dependent on subscriptions to carry out its undertaking.

Rules for Gratuitous Nursing of the Sick Poor.

1. All private staff nurses remaining in the Home, and not otherwise occupied, shall be engaged in nursing the sick poor, under the direction of the Matron in addition to the district nurses.

2. All recommendations for nursing to be made by a subscriber of not less than 10s. 6d., and countersigned by a minister of religion or by a medical man. A nurse will be supplied by the Matron at the earliest opportunity after receiving such recommendation. It is earnestly requested that in giving recommendation it shall be specially stated whether the case is infectious or not. Should a gratuitous nurse be sent to a case more than a mile from the Bargate, the subscriber or friend of the patient may be called upon to defray any travelling expenses incurred.

3. If a nurse is attending an infectious case, she must report it to the Matron, who will arrange that all her other cases be taken for her for the time being, and that she at the termination of such case be, with her clothes, properly disinfected at the Institute. During her attendance upon an infectious case she must on no account, except with the sanction of the Medical Officer, visit other patients.

4. The nurse will urge upon the patients and their friends the great importance of cleanliness in person and dwelling, of the immediate removal of all things offensive both from the bed and room, and of care to keep the sick-room clean and fresh at all times, of ventilation, of giving nourishment at the right time as directed, and of strict obedience to the orders of the medical attendant.

5. A supply of blankets, sheets, cushions and other necessaries is kept at the Home, to be let out on certain conditions which are arranged by the Visiting Committee.

6. No blankets, sheets, or cushions, or other like articles, to be lent until fully marked. The articles lent are to be registered by the Matron, with the date of the loan, and a corresponding list given to the responsible person receiving them; and such articles must be returned clean and in good order, at such times as the Matron may appoint.

### Cholera.

From the Nurse's Point of View.

Wiser heads than ours are responsible for the solving of the two great problems—(1) How to keep cholera out of England? (2) What to do when cholera creeps into our island? and we can safely leave those subjects to the men whose knowledge and experience qualify them for grappling with the insidious danger; but a few nursing details will not come amiss. The present appears to be a moment when our fellow-nurses can also do their part in personal preparation. They can take especial care to conserve, to build up in every reasonable way, their own bodily health. They can force themselves to pay strict attention to the necessity for plenty of fresh air, without too much accompanying fatigue. They can be conscientious in taking sensible nourishment, and they can be moderate in their indulgence in fruit. Now, we all know that fruit is a special temptation to nurses, and fresh fruit and good vegetables are certainly most agreeable parts of our summer dietary. Therefore, we do not say avoid these, but we merely give a modest warning, and urge that great discretion be exercised in the selection as well as in the quantity of such things. The Matron of one of the best nursing institutions in London, having had considerable personal experience of cholera patients, has for several weeks past been encouraging every private nurse, not required by the exigencies of the moment to stay in town, to take and to enjoy to the utmost possible limits a thorough holiday. A complete change into the most healthful possible surroundings, but to hold herself in readiness for a summons back to probably specially onerous duties by and bye. In our own probationer days a scare of cholera once arose, and, like all such subjects, the idea was hotly discussed in the nurses' sitting-room. Said one enthusiastic young lady, whose taste and capacity for work did not rank very high with her companions, "I shall volunteer to nurse any case that comes in. I should love to have a cholera patient." Older nurses looked up with grave surprise, but no one answered the rash words till the enthusiast asked, "Shouldn't you all want to go into the block they talk of holding in readiness next week?" Then a woman of much experience spoke, "My dear; I worked through an epidemic once, but I don't think I could speak as you do of a disease that killed half those who got it." At that moment the Matron entered the room, and after a few words of pleasant chat she said, "I know you are all thinking of the cholera. I have just been hearing the opinions of our physicians, and I am glad to say that most of them think it will not come to us this year. But if it should I may as well warn you now that I will allow no nurse, however complete her training, to go to the block unless she is in good mental and physical condition. It may be a fine field for good work, and for the display of high skill and unselfish devotion, but for emotional people, for those who are attracted by the sensationalism of novelty, there will be no openings, I assure you." The hot weather soon ceased, and the dreaded foe was kept out of London for that year, but many of us remembered her words, and unconsciously we grew to pay more attention to the removal of small ailments, and took greater care to preserve and to increase that stock of good health which is to everyone the best of all possessions. People in fair condition and amongst sanitary surroundings have little to fear, and those who nurse cholera patients have no need to dread "infection," except in the way in which that word also applies to typhoid fever; therefore it is not the personal danger she incurs which need have any weight with the careful attendant. Many of our nurses have never seen a case of cholera, even in the mild form. So much depends upon the nursing of this disease, and yet very few nursing handbooks mention it. The patient must be kept warm and recumbent, and medicines for the alleviation of pain are given promptly. The great danger is from collapse. Cramps can be alleviated by gentle rubbing. Raw meat juice in spoonfuls is generally the only food given. Mustard poultices may be ordered on the pit of the stomach to stay the vomiting. The patient should, if possible, be removed to a separate room, and only those in attendance should enter the room. The bed should be placed as much in the centre of the room as possible to enable the air to circulate freely. All hangings, curtains, and rugs must be removed. All the ejections of the patient should be immediately disinfected. This disinfection may be obtained by the addition of at least an equal quantity of a solution, containing to each quart two ounces either of chloride of zinc, sulphate of copper, or sulphate of zinc.

About a glassful of one of these solutions should be beforehand poured into the receptacles. All clothes, linen, sheets, blankets should, after use, be immediately soaked in a disinfecting solution.

The following are a few rules which must be implicitly obeyed by those attending to cholera patients. Neither food nor drink should be taken in a room occupied by a patient. The mouth should be carefully rinsed before each meal and the hands and forearms washed with a solution of borax. The face, head, and hands, and if possible the whole body, should be washed daily with water containing half an ounce of borax or fifteen grains of thymic acid per quart. And above all, let it be remembered that terrible as is a cholera outbreak, a cholera panic is ten times worse.

### Everybody's Opinion.

*[Correspondence on all subjects is invited, but we cannot in any way be responsible for the opinions expressed by our correspondents. No communications can be entertained if the name and address of the correspondent is not given, or unless one side of the paper only be written on.]*

### MALE NURSES.

"A Subscriber" writes: May I ask if there is any measure which can be taken to support properly-qualified male nurses, by inducing the medical profession to insist upon being supplied with really qualified men when sending to nurses' homes, &c., for them? In my opinion, few medical men know that men, who know little or nothing of the work entrusted to them, are sent out to patients requiring skilled attendance. I think you will agree that a grievance on this subject is justified on the part of those male nurses who have spent years in institutions to gain the experience which is necessary before going out "private nursing," whether it be to medical, surgical or mental cases; the latter I know most frequently necessitate male nurses, but all three are often nursed by men who are unqualified. May I ask your opinion on the subject?

### CAMBRIDGE HOME AND TRAINING SCHOOL FOR NURSES.

Miss Mary A. Young writes from the Nurses' Home, Fitzwilliam Street, Cambridge: In your very kind and most complimentary notice of the Cambridge Home for Nurses in your issue of 20th ult. there is one inaccuracy which I will ask you to correct. I am not indebted to Cambridge for my training, but have always been glad that I received it at King's College Hospital, London, when that hospital was nursed by the St. John's House Sisters, under whom I worked for three years. Subsequently I had charge of the Male Medical Ward at Addenbrooke's Hospital for nine years.

### PRIVATE NURSES.

"St. Helena" writes: It has been on my mind for some weeks to write to you anent an article in The Hospital treating of the training of nurses, and of private nurses in particular. You said, I think—for I quote from memory—that there are as yet few trained nurses in the proper sense of the word; that no nurse should be allowed to take private cases who had not had one year's training in a gene ral hospita containing not less than 100 beds, six months in midwifery, six months in massage, six months in fever nursing, and six months in district nursing. Now is not this rather overloading a nurse, the majority of whose cases may probably be of the quietest description? I know a little hospital of 30 or 40 beds where a nurse was sent temporarily from a private institution. She had had a three years' "training," and was chosen as being the "cleverest" nurse. Yet she said she had not seen in all the years of her private experience as much "real work" as in those three weeks. Another case might be mentioned of a nurse in a private house sent from another institution, also chosen as having had the "best training." The patient had some uterine inflammation and needed, besides douches, what nursing comfort and "complete rest" involved. Yet the patient was left with the bed clothes untucked at the foot at night, left for many hours to the servants while the nurse was out, and left in a direct draught while the nurse "dressed for the doctor." Yet this nurse was never tired of talking of the more showy "cases" she had had. Why, then, for instance, indispensably a hospital with "not less than 100 beds"? Are the opportunities and time for careful observation of cases to be met with in smaller hospitals not as valuable as some of the brain-dizzying rush of a larger one? Do we not rather want either different grades of nurses or a different standard altogether? I do not say our standard can ever be too high—"We needs must love the highest when we see it"—only, as Pilate asked, "What is truth?" one is tempted to ask, What is the highest? There are so many Sisters who think it more worth while to train the showy nurse than the one who is sober, honest, truthful, trustworthy, punctual, quiet and orderly, cleanly and neat, patient, cheerful and kindly, but "not brilliant." There are so many nurses already who consider their many accomplishments "lost" on so many of their private cases. I think it was in your own pages that words were quoted something to the effect, "We have medical women, literary women, political women: what we want is womanly women." I feel tempted to say: We have medical nurses, surgical nurses, technical nurses, scientific nurses: what we want is nurse-like nurses.

### Visitors in General Hospital Wards.

Settling down in a hospital, not as a patient, nor as an amateur philanthropist, but as a regular worker, society is not exactly what one looks forward most to acquire. But society, in its widest sense, is just exactly what we get, and moreover, we see humanity as it is, not as it generally appears to superficial observers, but that "plain unvarnished" truth which we are apt to think we want, but which in reality most of us weakly shrink from.

The patients who remain in for any length of time become our friends, and not only unfold to us the details of their own troubled and sordid lives, but exhibit a distinct interest in our special working existences. Perhaps it will show how strong this feeling of unity in interest often becomes, if we mention a queer old man who had been so grievously injured in a railway accident as to need months of residence in an accident ward. When he began to amend he was allowed to get up for a few hours daily, and then he took upon himself as many light duties as he could safely be trusted with. He distributed tea cups and criticisms with equal impartiality. He laid down very strict injunctions on manners whenever he thought a new or youthful patient inclined, however slightly, to transgress what he considered "proper" behaviour; but the height of his special supervision was reached when he was over-heard one morning solemnly warning a new importation in the nurse line, that she really must "look a bit sharper, and be more methodical," "because," he added, "we're very partickler here." It was rather a delicate position for the head nurse, who, however, exhibited her usual tact in convincing the well-intentioned old patient that a few points in ward jurisdiction were her sole prerogative.

Society, however, is not restricted to our patients, they are perhaps the most satisfactory ingredient in the whole, being naturally the centre of all our labours and the chief interest which binds together a somewhat heterogeneous household.

We have our fellow workers of both sexes, and we have our subordinates, not "inferiors," mark you! That unworthy term never occurs as an appropriate one to the people you know and associate with, those whose lots are cast in the dark places of the earth, and dwellers amongst the poor soon discard expressions which imply social superiority. Dark intellectually and literally are many of the crowded courts and loathsome rooms from whence the majority of hospital patients are gathered—and yet these men and women issuing from places in which the prosperous classes would scorn to kennel their dogs, behave with a decent patience and appreciative obedience which ought to surprise us, instead of being considered "only natural." They are generous to their fellow-patients, sharing ungrudgingly with the poorest; and higher courtesy still! they are civil to those idle wanderers through life, who, as a modern fashionable whim, have added hospital visiting to their other occupations (?) Perhaps this craze is one of the most rapid growths of the century, and fair ladies who a few years back did not know where Whitechapel was, and thought Shadwell and Walworth savage lands, now talk glibly to their neighbour at the dinner-table of their delight in visiting those "dear dreadful places, you know." And they give garbled accounts of some patient in a ward, whose eyes, or leanness, or what not, has stirred their fancy. Well, it hurts nobody but themselves. Seriously; we can comfort ourselves with that thought, but certainly no woman's character is elevated by making a pastime of "slumming." The word itself is vile, and should never have been fashioned. Would that it could be buried with all the unworthiness which it attaches to itself. When the poor are visited it should be done respectfully and gravely, with a view to helping them in the way most agreeable and suitable to their needs. To go to them in any other way, and for any other motive, is an impertinence. To patronise their poverty, to criticise their shifts, and to flatter the pretty and shrink from the plain amongst their children, by what right is this done? It is curious to see an elegant woman strolling through a ward full of sick children, and after ejaculating "How shocking" to each of the statements as to their ailments, conclude her survey and sum up her impressions thus: "Dear me, nurse, how very careless poor people are! All these accidents must be caused by negligence!" If the nurse, having the courage to express her own convictions, should say, "Yes, madam, the negligence begotten of that poverty which necessitates a whole family being housed in a single room; where saucepans of boiling water get put on the floor, because there is no other place for them, and the children get scalded, and the babies get burnt to death, because they have to be left in charge of other mere babes whilst the mother is out at work, and the delicate infants die because they sleep and live in poisoned air, and the strong ones grow weakly through improper and insufficient feeding. Yes, madam, it is negligence; but *whose* we shall know better some day, only *don't* judge the mothers too hardly. Should you or I do better, or as well, if we had their burdens to bear?"

Probably our fine lady tells her friends, "The nurse spoke to me in the most extraordinary way to-day! Really, she was hardly civil. I think I shall speak about it next time I go to the hospital; it ought to be inquired into."

Another visitor wonders how poor people can bear to see their children so dirty, and it is hard to convince her that one basin, with a large piece out, is often the only apology for a bath in a family. And although the pail, which does for many other purposes, or even a washing-tub may be amongst the properties, a small kettle is the sole means by which water, fetched from the basement, can be heated for the family ablutions.

Cleanliness becomes a luxury difficult of attainment amidst such drawbacks as these. Comparative dirt and comparative purity are possibilities, but spotlessness is not to be hoped for, and must not be required until we house our poor better, and then educate them to live up to a higher standard.

Other members of our hospital society are always regarded with a little awe, which does not often surround the ignorant section of the prosperous public, for they are, or at any rate are supposed to be, experienced in our ways and wants. We refer now to members of the council or committee of management, and some of them are splendid, practical, liberal men, but others are not. They visit the wards either regularly or irregularly as they please, and they are apt to fancy themselves omnipotent because the regular workers, whose salaries and maintenance come within their jurisdiction are, so to speak, in their power. This fact gives a pleasant sensation of beneficence to the well clad gentleman who strolls round the wards making a perfunctory inspection, congratulating a sister on the fashionable hue the new paint gives to her walls or praising a handsome plant, a gift, but not of his providing.

These committee people might be very useful, but they seldom are; instead of aiding the officials they often hinder them, chiefly because they do not work in the right way or the just spirit. They should encourage free speech, but not promote discontent; they should let all workers feel that they can rely upon fair treatment, and that no person need fear being made a scapegoat if he or she makes reasonable reports to the proper quarter of remediable evils. Those who feel that grievances, real, not imaginary ones, are sure of redress, will rest content with management which shows that the protection of the interests of the employed is recognized as an important feature in hospital jurisdiction.

### Some Australian Experiences.

(*Continued from page* clviii.)

I WAS awakened next morning by the children's voices outside, and after breakfast, I made their acquaintance. Very happy they all were too; we were soon on the best of terms, and I promised to go and see all their puppies and kittens, which were very numerous, the kittens and cats in particular; the reason given is, that where there are plenty of cats there are so few snakes. Later on, I went for a confidential chat with my patient, and very minute and elegant were all the preparations for the little stranger. No lady residing within shopping distance of London could have had things more fitting the occasion, or a more comfortable bedroom. After this interview, I fulfilled my promise to go round the place with the children. I was both surprised and delighted. First we went all over the house, which was on the ground floor, and all the principal rooms lead out on to the verandah. Lovely dining, drawing, breakfast, and best bed-rooms constituted the main building; the kitchen, pantry, and servants' bed-rooms were in a separate block; then the laundry, larder, and dairy, and also another store-room, and the children's school-room was apart from the rest. Then there are the "barracks," where the single men of the "station" live and sleep, and have a cook to themselves; the stables and out-houses are also inside the homestead enclosure. At the front of the place there is a lovely flower garden with orange, lemon, peach, almond, and other fruit trees planted in groves at the rear, and a large vegetable garden. As the "Home-stead" is built on the banks of the Murrumbidgee river, there is always a good supply of water, pumped up by a windmill on the bank. Outside the "Homestead" fence is the "sheep run," the miles and miles of plains spoken of before, and at certain distances apart are the shepherds' huts—very lonely places, too, for as these "runs" are sometimes miles in extent, so are the huts apart, and consequently it is sometimes weeks together that the shepherds spend without seeing a human being, especially when the grass gets scarce, and they have to "travel the sheep" farther back for feed and water. Sometimes these poor fellows become quite imbecile from sheer loneliness. About a mile or so from the "Homestead" is the shearing shed, and all the paraphernalia for sorting, pressing, and packing the wool. As the "shearing" was in full swing, of course the children invited me to accompany them after school; so off we went. It was most interesting. I tried my hand at shearing, but after cutting a piece out of a poor sheep, gave it up. The men used to quite look forward to our visits. The discipline of the shed is splendid. At a given signal, all hands cease work, say for dinner, tea, or for smoking, which is not allowed inside. I was generally there for afternoon "Smoke oh." One man calls out "Smoke oh," then all stop for a smoke, then a pail of tea without milk is hung up, and each man dips in his "pannikin." A large dish of nice little cakes called "brownie" comes with tea, so we generally partook of that. These men although so rough, are very particular about their food, and pay their camp cook well. Adjoining the wool shed are the shearers' huts, fitted up inside with bunks just like on board ship; they roll themselves in their blankets and are very comfortable. I was there six weeks, and my patient having done very nicely, I quite enjoyed the experience, it was all so different to what one would expect. Although so far from the capital, they dine late, dress for dinner, and keep up all the usual rules of a town house, and keep a staff of servants from the butler to the kitchenmaid—so all life in the "Bush" is not rough. The children had a governess to teach them. There is plenty of variety, too, and numbers of visitors. People travelling from one part to another, call and stay at the "station," from a regular town gentleman to an old "sundowner," *i.e.*, a man who generally contrives to reach a "station" at sundown, and claims rations and shelter for the night. Many men get over hundreds of miles in this way. It is always a good plan to carry your riding-habit, as horses are nearly always available, and a good ride through the Bush or across the plains is most enjoyable, and blows away the cobwebs. I always got plenty of riding wherever I nursed in the country.

After my return from the "sheep station," I entered the Prince Alfred Hospital, Sydney, as ward "sister," and as I have been asked so many questions about doctors, nurses, and hospitals in Australia since my return, I will try my best to describe hospital life as I found it there, and I think it will compare very favourably with hospital life in England, and also show that "anything" does not do for the Colonies, but, on the contrary, a few hints might be gained, considerably to the advantage of our "home" nurses. In the first place, the hospital itself is a very handsome brick and stone building, after the style of St. Thomas's Hospital, London, constructed in pavilions, connected by bridges, and sheltered by broad verandahs. At the time I speak of, the principal pavilions were C and D, the former top wards being the female medical, the lower for the female surgical, eye, and children's, while D contained male medical, male surgical and accident. Since then eye, children's, and convalescent surgical wards have been opened. Each of these wards contains 32 beds, always full. There is the sister, the head nurse, the assistant nurse, and two probationers to each of these four wards, no ward maid, but a woman to scrub the floor once a week, all the rest ofthe work is done by the nurses. Attached to each ward is a ward kitchen, private ward, "Sister's" room, private bath-room, bath-room and lavatories for patients. All these are fitted up in the newest style, so there is plenty of brass work, &c., to keep bright, and very bright it is. All the kitchen and ward utensils are either copper, brass, or tin, and are cleaned every morning.

(*To be continued.*)

### Presentation.

LONDON LOCK HOSPITAL AND ASYLUM, HARROW ROAD.—The nurses of the Female Lock Hospital have presented Dr. Kingston, who has for over two years been the senior house surgeon at the hospital, with a silver cigarette case and match box, as a token of gratitude for his great kindness to them. Dr. Kingston is leaving the hospital to go into private practice.

### Notes and Queries.

### Answers.

*C. F.*—Quain's Anatomy or Mivart's Elementary Anatomy.

*A. M. C.*—It is very unwise for any nurse to go abroad now on mere chance. Watch the advertisements and inquire privately, or write to the hospitals at the Cape if that is the colony you wish to go to particularly.

*Dispenser, Coleraine.*—Your query is vague; you do not say where you want to learn, or whether you want to pass the School of Pharmacy of the Pharmaceutical Society of Great Britain. Miss Swain, the resident Lady Dispenser at Warneford Hospital, Leamington, takes pupils, ten guineas for six months' tuition. The Society of Apothecaries' fee for examination is two guineas. A qualified chemist could teach you provided you could pass the examination after. The Pharmaceutical Society in Ireland teaches dispensing to women. You had best write to them, but if this does not tell you what you require, write to "Nursing" at this paper.

*Nina.*—Three years' training in a good provincial hospital where certificates are granted would serve your purpose. If you prefer London write to the matrons of King's College Hospital, the Royal Free, Gray's Inn Road, and London Hospital, Whitechapel, asking for forms of application for probationers. If you require further information or advice, pray write to us again

WE have received a communication from "A Late Sister" of the Birmingham General Hospital, which lack of space prevents our inserting. We are pleased to read her cordially-expressed testimony to the various improvements in the nursing arrangements which have taken place during the late Matron's seven years of office.

### "The Flowers of Sleep."

*(Concluded from page clx.)*

It was in the autumn term of the same year, a Saturday afternoon. The pupil teachers were assembled in the playground of the D—— Street Board School—a piece of elevated land on the outskirts of the town.

The afternoon was lovely. Above the sky was so dark, and so blue, and so intense beside the frail white clouds roaming through it, that the eye turned for relief to the gold mist spreading over the hills from beneath a rack of dark clouds trailing along the horizon.

"You here, Mr. Carew?" said Miss Pierce in great surprise, as he rose from the bench where he was sitting to shake hands. "I believe this is the first time you have ever come up, though we opened last June."

"Yes, it is the first time," he replied. He was looking very ill. "One sees so much of these faces during the week that one is glad to escape from them for a short space."

Violet did not sympathise with this view, so she remained silent. Much as she liked him, much as she felt for him, she could never think of anything to say when they were alone together. After a few meaningless remarks about the game they were watching, and a few awkward pauses, it was with a feeling of relief that she noticed Rachel coming across the fields to the playground with a basket of poppies on her arm.

The master's whole face changed. "May I speak to your friend?" he asked eagerly.

Miss Pierce looked at him in surprise. Perhaps she guessed the reason why he had ventured to the tennis-court that day. "I will introduce you to her," she said half-sadly.

Rachel came over to them with her light swinging step, her face almost sublime after the long lovely day amid the woods and hills.

After a little conversation Violet introduced the two, and went to join a set.

"What lovely flowers!" said Carew, presently.

"Are they not exquisite? I have been walking all day among sloping fields of corn glinting with poppies."

"I generally spend my Saturday afternoons among the hills," he remarked. "How wonderful it is when waves of wind sweep over them, and the corn turns a darker hue, and the poppies burn up like fire!"

"That is in the sun. In the shade what impresses you most is the silken texture of each individual flower, and its form, the slender stem, the great loose leaves."

He pointed to her poppies. "It is strange that these brilliant hues of life and this divine form are connected in our minds with sleep and with death," he said, thoughtfully.

Rachel had altered her first impression about Carew. She now felt his must be a beautiful and original mind, she pitied him from the bottom of her heart.

"It is hard for sleep as for death to exist even in thought on such a day as this," she replied; "sometimes they seem so far off."

"They are always so near," he murmured.

Rachel looked at him with calm, critical eyes. "Excuse me," she remarked, "but you have been overworking—you are looking very ill. You find teaching a strain?"

"It is extremely wearing, I do not like it," he replied; "but I write a great deal as well, and perhaps I attempt too much."

"What do you write?" she asked.

"Verse, generally," he said. He had no difficulty in speaking to her, and again the feeling of hope came over him.

"Have you published anything?"

"No, not yet. I write for the intense joy it gives me; some day I hope to bring out a volume."

"I wish you all success," said Rachel, sympathetically, "but you must not overdo yourself. You must take the greatest care of your health if you wish to give your brain a fair chance."

"I will, indeed I will," he replied.

At that moment the Superintendent strolled into the playground. Rachel knew him intimately by this time, and after a little conversation she took him aside to speak to him about Carew's health.

"Poor fellow! Yes, he is breaking down," said Atkins, "and he's doing no good at the school."

"He must have a rest," said Rachel emphatically, and they talked the matter over for a long time together. Atkins said Carew would not care to take up teaching again if he left the classes, and Rachel thought her father might help him to a post as clerk, where the work would at least be mechanical and free from responsibility. Then the Superintendent spoke to her of something else—of his love for her. There was an undercurrent of romance beneath his plain and practical words. She liked him very much, but to-day there was a momentary hesitation in her mind before she gave her answer. As he returned to the tennis-court, Carew watched the two from his bench. "It is foolish, very foolish, to mind so much!" he cried bitterly to himself, pressing his hands together. Then the sun seemed to glare upon him suddenly from behind the clouds—a giddiness came over him—he lost consciousness for a moment . . . . and he found Violet Pierce standing beside putting a cup of cold water to his lips.

"What happened to me?" he asked, bewildered.

"You are ill, you fainted," she replied, "you must see a doctor."

"It was only the scent of the poppies—it dazed me," he said, adding half-sadly in a lower voice, "It is the beginning of the end. They are truly called the flowers of death."

Towards the end of the Christmas holidays Rachel McClure and Walter Atkins were walking down Oxford Street. He was staying at her father's house. They were to be married shortly.

"So you will have nothing but books for your wedding present?" asked the Superintendent. "Well, we have a couple of hours to ransack all the shops. Here's a promising one to begin with. May I have your list?"

Rachel produced a somewhat lengthy list of books she wanted to look over before buying; they went into the shop and spent a most delightful half-hour inspecting all the new editions.

"Would you like to see this volume of 'Poems' just out? It has been most highly reviewed in the papers," said the Assistant, handing Rachel a small dark-blue book with the design of a large drooping poppy in red on the cover.

She opened it at the title-page, and gave a cry of surprise. "'Poems, by Gilbert Carew.' I am so glad he has brought them out!"

"Gilbert Carew, and Poems," exclaimed Atkins, "I cannot connect the two ideas."

"He told me that he wrote," replied Rachel, delightedly, turning over the leaves. She paused suddenly to read a lyric called "The Flowers of Sleep"; about poppies; about what he had said to her that day in the playground. It was so beautiful and so sad that the tears came to her eyes. She wished she had seen more of him; she might have helped him—inspired him.

"And you say they have been well spoken of?" asked Atkins.

"Yes, sir, everywhere. Poor young man! Such a sad ending."

"How?" cried Rachel, terrified.

"Haven't you seen the notices in to-day's papers? They're full of it."

"His death?" suggested Atkins, growing very white.

"What do you mean?" said Rachel, with a sense of sudden darkness.

"Here is the paper, sir. They say he was not strong enough for teaching-work. A young man of such promise, too!"

Rachel took the paper and read.

"Violet loved him, and I might have loved him," she said to herself, "we should have saved him."

Ethel Wheeler.

## Figures and Tables

**Figure f1:**